# MicroRNA expression in pre-treatment plasma of patients with benign breast diseases and breast cancer

**DOI:** 10.18632/oncotarget.25262

**Published:** 2018-05-11

**Authors:** Mirelle Lagendijk, Sepideh Sadaatmand, Linetta B. Koppert, Madeleine M.A. Tilanus-Linthorst, Vanja de Weerd, Raquel Ramírez-Moreno, Marcel Smid, Anieta M. Sieuwerts, John W.M. Martens

**Affiliations:** ^1^ Department of Surgical Oncology, Erasmus MC Cancer Institute, EA 3075, Rotterdam, The Netherlands; ^2^ Department of Medical Oncology, Erasmus MC Cancer Institute, EA 3075, Rotterdam, The Netherlands; ^3^ Cancer Genomics Centre Netherlands, Erasmus University MC, CN 3015, Rotterdam, The Netherlands

**Keywords:** microRNA, breast cancer, BRCA1-mutation carriers, micro array, RT-qPCR

## Abstract

**Background:**

MicroRNAs (miRs) are small RNA molecules, influencing messenger RNA (mRNA) expression and translation, and are readily detectable in blood. Some have been reported as potential breast cancer biomarkers. This study aimed to identify and validate miRs indicative of breast cancer.

**Results:**

Based on the discovery and literature, 18 potentially informative miRs were quantified in the validation cohort. Irrespective of patient and tumour characteristics, *hsa-miR-652-5p* was significantly upregulated in the malignant compared to benign patients (1.26 fold, *P* = 0.005) and therefore validated as potential biomarker. In the validation cohort literature-based *hsa-let-7b* levels were higher in malignant patients as well (1.53 fold, *P* = 0.011). Two miRs differentiated benign wildtype from benign *BRCA1* mutation carriers and an additional 8 miRs differentiated metastastic (*n* = 8) from non-metastatic (*n* = 41) cases in the validation cohort.

**Methods:**

Pre-treatment plasma samples were collected of patients with benign breast disease and breast cancer and divided over a discovery (*n* = 31) and validation (*n* = 84) cohort. From the discovery cohort miRs differentially expressed between benign and malignant cases were identified using a 2,000-miR microarray. Literature-based miRs differentiating benign from malignant disease were added. Using RT-qPCR, their expression was investigated in a validation cohort consisting of pre-treatment benign, malignant and metastatic samples. Additionally, benign and malignant cases were compared to benign and malignant cases of *BRCA1*-mutation carriers.

**Conclusions:**

Plasma microRNA levels differed between patients with and without breast cancer, between benign disease from wildtype and *BRCA1*-mutation carriers and between breast cancer with and without metastases. *Hsa-miR-652-5p* was validated as a potential biomarker for breast cancer.

## INTRODUCTION

Early diagnosis improves breast cancer survival [1]. National screening programs aim to detect breast cancer at an early stage. Woman aged 50–75 are invited for biennial screening with additional annual screening programs for patients with a cumulative life time risk of breast cancer of over 20% (40–50 years) and BRCA1/2-mutation carriers (25–75 years). Mammography and breast MRI (BRCA1/2-mutation carriers) are the radiological modalities used in these programs. Mammographic sensitivity is, however, generally impaired in patients with dense breast tissue found especially in the younger population [2]. In the national screening program performed in the general population 20% of the carcinomas is detected between two screening moments [3]. Improved sensitivity of screening could possibly reduce the number of these interval carcinomas. Improved specificity could diminish distress as a result of false-positive (mammographic) result. Biomarkers detectable in body fluids could be valuable as this allows for minimally invasive screening and repetitive monitoring [4].

Blood-based microRNAs (miRs) are potential biomarkers for breast cancer. MiRs are small RNA molecules that influence regular messenger RNA (mRNA) expression and translation by binding with (partially) complementary sequences on target mRNAs aided by the RNA-induced slicing complex [5]. The resulting post-transcriptional gene modulation can lead to mRNA degradation, translational inhibition or translational activation [6]. MiRs, as opposed to mRNAs, are extremely stable under different conditions and readily detectable in serum and plasma [7].

In breast cancer, multiple miRs have been associated with oncogenic driver events, cancer invasiveness, adverse clinical outcome and therapy resistance [5, 8–12]. Most of these studies used tissue samples whereas blood-based markers are needed for screening purposes. Previous studies focusing on miRs present in the circulation (serum or plasma) showed that the quantity and composition of blood-based miRs can predict the presence of tumour [7, 9, 13, 14]. Differences in miR expression levels between breast cancer patients and healthy controls enables the use of miRs as a blood-based diagnostic biomarker.

This study aimed to identify and validate miRs indicative for breast cancer in plasma of patients prior to surgery who did not receive neo-adjuvant treatment with a long follow-up term. The cohort consisted of patients without and with a family history of breast cancer including BRCA1-mutation carriers. MiRs discriminative for benign and malignant breast disease in a discovery cohort and miRs reported in literature to be differentially expressed between breast cancer patients and healthy controls [15–21] were validated in an independent validation cohort.

## RESULTS

One discovery sample (3.3%) was excluded based on a high haemolysis score resulting in 29 suitable samples. In the validation cohort 4/84 (4.7%) and 1/84 (1.2%) samples were excluded based on a too low expression level of the 5 reference miRs and/or a high haemolysis score, respectively, resulting in 79 suitable samples. Baseline characteristics are specified in Table 1. The two cohorts were well balanced considering the clinical parameters. The median follow-up time was shorter in the discovery cohort than the validation cohort: 9.2 [interquartile range (IQR) 7.7.-10.1] versus 11.0 [IQR 7.4-11.8] years, *P* = 0.043. Breast cancer metastases occurred in 6 (32%) and 8 (16%) of the breast cancer patients during follow-up with a median time since blood sampling of 0.9 [IQR 0.3-4.4] and 2.1 [IQR 0.3-4.4] years for the discovery and validation cohort, respectively, *P* = 0.53. Local breast cancer recurrence was not reported in either cohort during follow-up.

**Table 1:** Clinical characteristics for the discovery and validation group

All	Discovery	Validation	*P*-value
	(*n* = 29)	(*n* = 79)	
**Age** median in years (min-max) ^¥^	49.9 (39.7–55.2)	55.3 (48.9–61.5)	0.07
**Follow up** median in years (min-max)^¥^	9.2 (7.7–10.1)	11.0 (7.4–11.8)	0.043
**Classification all samples**^^^			0.37
Benign	10 (34%)	30 (38%)	
Non-metastatic BC	13 (45%)	41 (52%)	
Metastatic BC	6 (21%)	8 (10%)	
**Classification BRCA1-mutation carriers**	(***n* = 5)**	(***n* = 3)**	
Benign	3	1	
Non-metastatic BC	1	2	
Metastatic BC	1	0	
**Classification familial breast cancer**^^^^	(***n* = 6)**	(***n* = 20)**	
Benign	0	8 (40%)	
Non-metastatic BC	4 (67%)	10 (50%)	
Metastatic BC	2 (33%)	2 (10%)	
**Breast cancer patients**	(***n* = 19)**	(***n* = 49)**	
**Tumour stage**^^^^			0.26
T1	9 (47%)	28 (57%)	
T2	7 (37%)	19 (39%)	
T3	3 (16%)	2 (4%)	
**Nodal stage**^^^^			0.12
N0	5 (26%)	25 (51%)	
N1	10 (53%)	16 (33%)	
N2	3 (16%)	4 (8%)	
N3	0	4 (8%)	
Unknown	1 (5%)	0	
**Oestrogen receptor status**^^^			0.51
Negative	5 (26%)	9 (18%)	
Positive	14 (74%)	40 (82%)	
**Her2Neu receptor status**^^^^			0.73
Negative	17 (89%)	33 (67%)	
Positive	2 (11%)	9 (18%)	
Unknown	0	7 (14%)	
Triple negative breast cancer^*^^^^^			0.22
No	14 (74%)	43 (88%)	
Yes	5 (26%)	5 (10%)	
Unknown	0	1 (2%)	

BC - Breast Cancer. ^¥^ Mann–Whitney *U* test ^Fishers exact test with a Freeman-Halton extension when appropriate (2-tailed *P*-value). ^^Kruskall Wallis test. ^*^ER-receptor, PR-receptor and Her2Neu receptor negative breast cancer.

### Discovery phase

**Figure 1:**
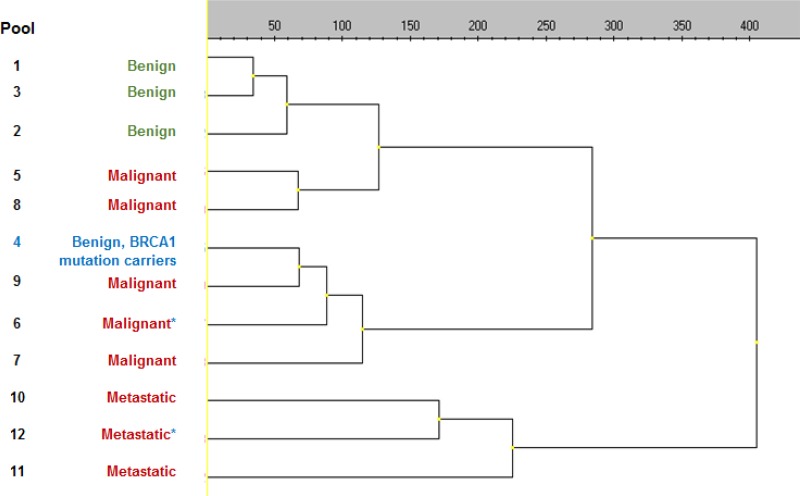
Dendrogram unsupervised hierarchical clustering discovery cohort Clustering of the 12 groups present in the discovery cohort, based on the expression of *n* = 756 expressed miRs, was performed with the software package of GenEx v.4.1.76.1 from MultiD. Clustering method: Ward's Algorithm. Distance measure as indicated above the dendogram: Euclidean. ^*^Groups which contain BRCA1 mutation carriers.

The unsupervised analysis of the 756 miRs detected with the microarray clearly showed a separated hierarchical clustering of the benign wildtype *BRCA1* and the other benign samples (Figure 1). The benign *BRCA1*-mutations carrier samples, however, clustered with the malignant cases.

**Figure 2:**
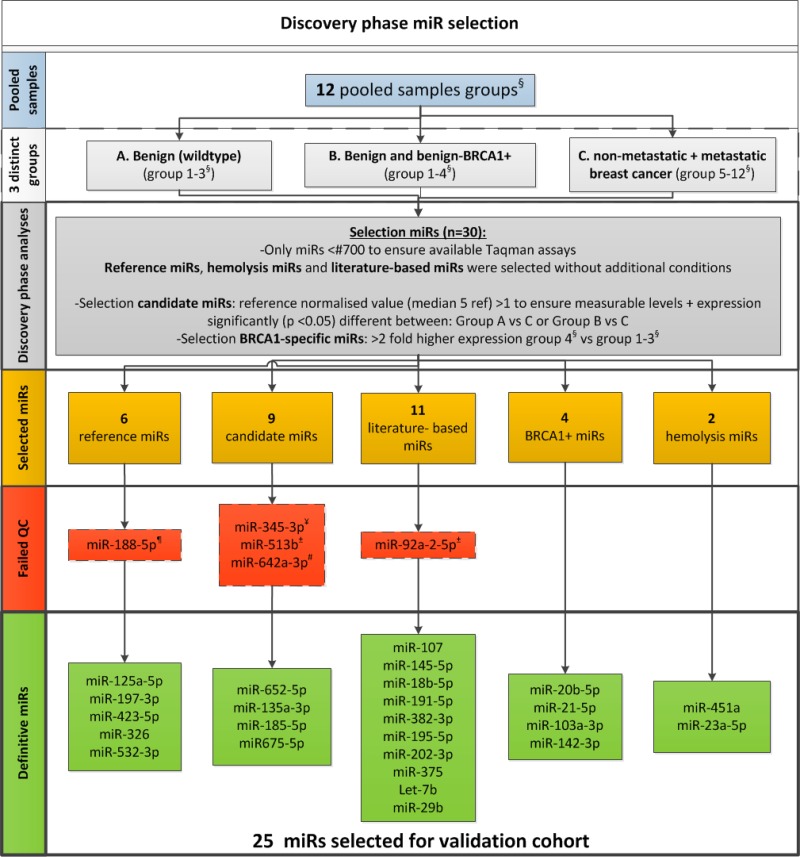
Discovery phase flow-chart ^§^Supplementary Table 2 displays a detailed description of the pooled groups. ^*^Mann-Whitney *U* test. QC = Quality control. ^¶^Too high expressed in multiplex vs uniplex; appears to detect additional transcripts. ^¥^Poor amplification and too low expressed for a reliable analysis. ±Too low expressed for a reliable analysis. ^**^Poor amplification curves and poor efficiency in multiplex.

Of the 756 miRs, 7 miRs were significantly differentially expressed between benign and malignant pooled samples and were therefore selected for validation (Supplementary Table 1). Since the benign *BRCA1*-mutations carriers samples, clustered with the malignant cases, 4 additional miRs most discriminatory between benign *BRCA1*-mutation carriers and wildtype samples were added to our selection to further investigate this observation (Figure 2 and Supplementary Table 1). In addition, 11 literature-based miRNA and 6 stable expressed reference miRs selected from the microarray were included for analysis in the validation cohort (Supplementary Table 2). Twenty-five out of 30 miRs (5 reference miRs for quantification, 2 haemolysis markers and 18 candidate miRs) passed our quality control steps with respect to reproducibility and PCR efficiency (Figure 2). The 18 candidate miRs were quantified in the validation samples by RT-qPCR using validated Taqman mature MicroRNA Assays (ThermoScientific).

### Validation phase

#### Benign versus malignant

**Figure 3:**
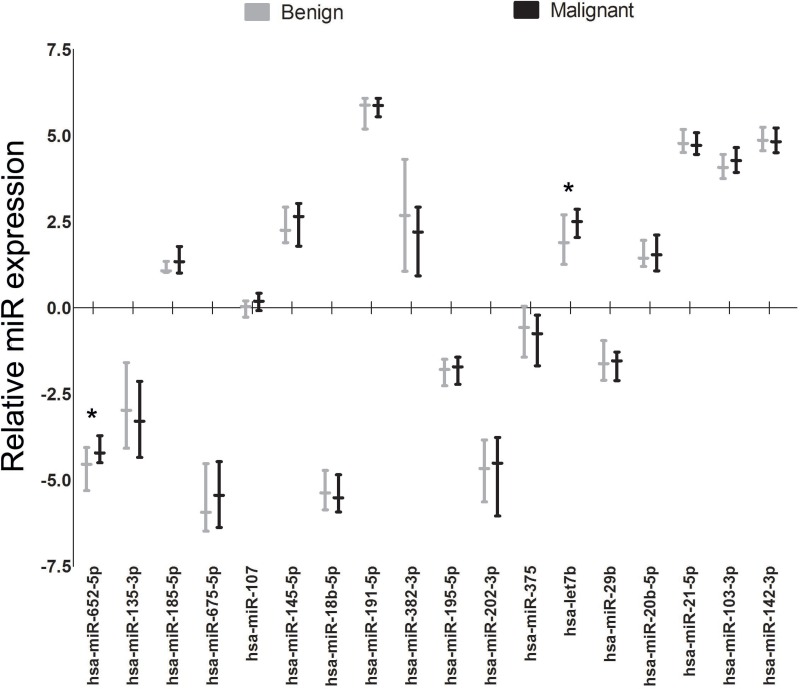
Relative miR expression of candidate miRs for malignant versus benign validation samples The y-axis reflects the relative miR expression as compared to the overall expression of the 18 miRs evaluated in the validation cohort. The median and interquartile ranges per miR are displayed by the box-plot. ^*^P-value< 0.05 (Mann–Whitney *U* test, after correction by Benjamini-Hochberg method (10% FDR).

*Hsa-miR-652-5p*, a selected candidate-miR in the discovery cohort, was significantly upregulated in the validation cohort and therefore validated as a potential biomarker for the detection of breast cancer. Has-miR-652-5p was statistically significant upregulated in the malignant samples (*n* = 49) compared to the benign samples (*n* = 30), fold change 1.26 (*P* = 0.006) (Table 2 and Figure 3). *Hsa-let-7b,* a literature-based miR, also was statistically significant upregulated in malignant samples compared to benign samples, fold change 1.53 (*P* = 0.011) (Table 2 and Figure 3). Neither *hsa-miR-652-5p* nor *hsa-let-7b* showed a significant correlation with age (Spearman’s’ rank correlation coefficient −0.044 (*P* = 0.77) and 0.169 (*P* = 0.257), respectively). Within the *n* = 49 breast cancer cases, no statistical differences in the expression levels of both miRs were found regarding tumour stage, nodal status, hormonal receptor status, HER2 status or triple negative breast cancer (TNBC) status (data not shown).

**Table 2:** Differentially expressed (relative expression) validation cohort

Clinical group	All			Malignant		
	*malignant (49) vs Benign (30)*			*Metastatic (8) vs Non-metastatic (41)*		
miR	Fold change^1^	*P*-value^*^	*P*-value^**^	Fold change^2^	*P*-value^*^	*P*-value^**^
*hsa-miR-18b-5p*	0.91	>0.05	>0.05	0.52	0.028	0.014
*hsa-miR-21-5p*	0.96	>0.05	>0.05	0.50	0.022	0.014
*hsa-miR-29b*	1.06	>0.05	>0.05	0.71	0.044	0.022
*hsa-miR-135-3p*	0.80	>0.05	>0.05	1.89	0.017	0.008
*hsa-miR-195-5p*	1.06	>0.05	>0.05	1.02	0.040	0.019
*hsa-miR-202-3p*	1.11	>0.05	>0.05	0.48	0.033	0.017
*hsa-miR-382-3p*	0.72	>0.05	>0.05	0.09	0.011	0.006
*hsa-miR-652-5p*	1.26	0.006	>0.05	0.99	>0.05	>0.05
*hsa-miR-675-5p*	1.41	>0.05	>0.05	1.36	0.006	0.003
*hsa-let7b*	1.53	0.011	>0.05	0.91	>0.05	0.031

^1^Fold change (malignant/benign). ^2^Fold change (metastatic/non-metastatic).

^*^Comparison between groups using the Mann–Whitney *U* test. ^*^P-value based on correction by Benjamini-Hochberg method (10% FDR). ^**^P-value based on correction by Benjamini-Hochberg method (5% FDR).

### BRCA1-mutation carriers

The 4 miRs selected in the discovery cohort based on a 2-fold higher expression in the benign BRCA1-mutation carriers versus wildtype benign cases showed no differential expression in the validation cohort (data not shown). When investigating expression in the individual benign samples (discovery and validation cohort) of *BRCA1*-mutation carriers (due to the low numbers, discovery and validation cohort combined) with wildtype benign samples (validation cohort) both *hsa-miR-185-5p* and *hsa-miR-675-5p* showed an differential expression with a fold change 2.16 (*P* = 0.005) and 0.25 (*P* = <0.001), respectively (Table 3). No differences were found comparing malignant *BRCA1*-mutation carriers (discovery and validation cohort, *n* = 3) to wildtype malignant samples (validation cohort, *n* = 39) (data not shown).

**Table 3:** miR expression for BRCA1 vs non-BRCA1 samples

Clinical group	Benign		
	*BRCA1 benign (4) vs Non-BRCA1 benign (29)*		
miR	Fold change	*P*-value^*^	*P*-value^**^
*hsa-miR-185-5p*	2.16	0.005	>0.05
*hsa-miR-675-5p*	0.25	<0.0001	0.003

Fold change (wildtype/BRCA1 mutation carriers).

^*^Comparison between groups using the Mann–Whitney *U* test. ^*^P-value based on correction by Benjamini-Hochberg method (10% FDR). ^**^P-value based on correction by Benjamini-Hochberg method (5% FDR).

### Malignant; non-metastatic versus metastatic breast cancer

**Figure 4:**
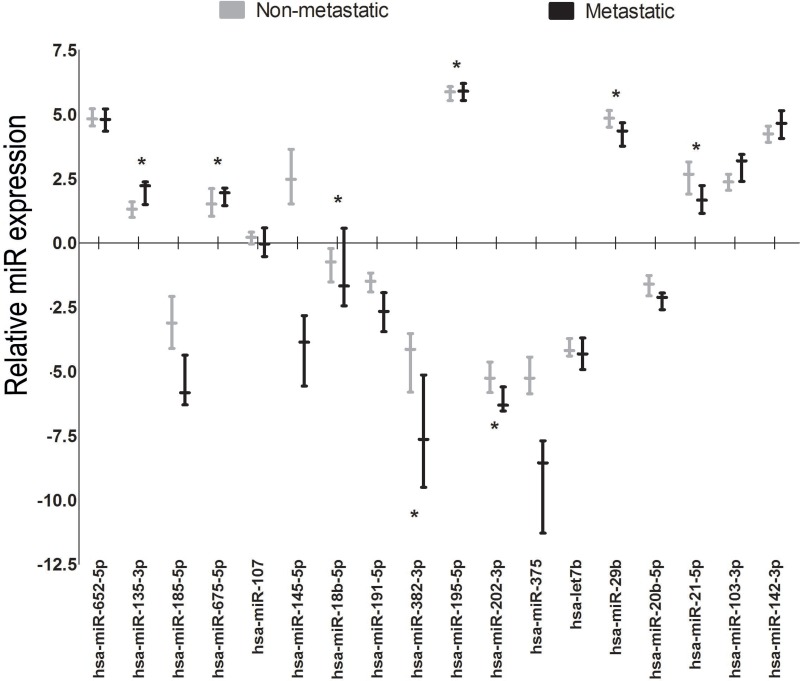
Relative miR expression of candidate miRs for metastatic versus non-metastatic validation samples The y-axis reflects the relative miR expression as compared to the overall expression of the 18 miRs evaluated in the validation cohort. The median and interquartile ranges per miR are displayed by the box-plot. ^*^P-value< 0.05 (Mann–Whitney *U* test, after correction by Benjamini-Hochberg method (10% FDR).

Eight out of 49 (16.3%) breast cancer patients were diagnosed with metastatic breast cancer during follow-up (Table 1). Comparing metastatic to non-metastatic breast cancer samples *hsa-miR-135a*, *hsa-miR-195-5p* and *hsa-miR-675-5p* showed an upregulated expression whereas *hsa-miR-18b-5p*, *hsa-miR-21-5p*, *hsa-miR-29b, hsa-miR-202-3p* and *hsa-miR-382-3p* showed a downregulated expression in metastatic samples (Table 2 and Figure 4).

## DISCUSSION

MiRs can be useful biomarkers and we identified one, *hsa-miR-652-5p*, for which pre-treatment plasma levels are different between malignant and non-malignant disease. The higher expression of this miR in breast cancer patients was confirmed in an independent validation cohort. As the expression did not correlate with either patients’ age, tumour stage, nodal stage or hormonal receptor status and Her-2/Neu status, *hsa-miR-652-5p* may be widely applicable as a possible blood-based biomarker for breast cancer detection. The use of a miR signature is, however, preferred over the use of a single miR as a potential biomarker since a combination of miRs usually improves diagnostic accuracy [17, 25, 26]. In the current cohort only miR *hsa-miR-652-5p* was validated as a potential biomarker. In line with these results, *hsa-miR-652* has previously been reported as a breast cancer biomarker with a upregulated expression comparing benign breast diseases to breast cancer patients [26] and healthy controls versus benign and versus malignant breast diseases [27]. As in our cohort, both previously published studies showed an upregulated expression irrespective of tumour stage, thus supporting the use of *hsa-miR-652* as a potential biomarker for early stage breast cancer detection. To the best of our knowledge the genome context or target context of *hsa-miR-652-5p* has not been described previously.

The literature based candidate *hsa-let-7b* was also significantly differentially expressed between benign and malignant samples within the validation cohort. The *let-7*-family is associated with multiple oncogenes and upregulated *let-7*-levels have been associated with a poor prognosis and poor overall survival [20, 28]. The upregulation observed is in line with other studies showing an upregulated expression in breast cancer patients [17, 28, 29] with higher levels in metastatic breast cancer [29]. Higher levels of *hsa-let-7b* between metastatic and non-metastatic patients were, however, not observed in the current cohort. The validation cohort, however, did show 8 miRs that were significantly different in breast cancer patients with metastasis compared to those without. These miRs could possibly be used to predict the onset of breast cancer metastasis. Although some of these miRs have been described as possible biomarkers differentiating between healthy and malignant samples [15, 18, 19, 30, 31], only the downregulation of *hsa-miR-29b* has previously been reported to differentiate non-metastatic from metastatic breast cancer samples [31]. When performing an additional sensitivity analysis comparing the individual non-metastatic (*n* = 13) to metastatic samples (*n* = 6) of the discovery cohort no significant differences were found in miR expression levels. This can possibly be explained by the limited patient number available in the discovery cohort and overall small differences in absolute miR expression levels found in both cohorts. Therefore, independent validation of these miRNAs in a large cohort is needed.

Although promising as potential biomarkers, several methodological issues have to be resolved to take miR expression data to the clinical arena. Multiple techniques are described for miR detection and selection or the type of normalisation of miR-data, possibly influencing the results or interpretation. The range of the miR expression level is influenced by the normalisation on which a relative expression or fold change is calculated. The normalisation of the expression levels varies between studies potentially hampering validation of previously obtained results. In the search for a potential breast cancer biomarker differences in clinical characteristics influencing miR expression levels should be accounted for when comparing cohorts. As shown in multicentre cohorts harbouring breast cancer patients, differences in MIR expression are also present between patients with benign breast disease(s) and healthy controls [25, 26]. In a large cohort, Shimomura *et al.* underlined the wide variation in miR expression levels present within the healthy population [25].

The strength of the current study is the use of a validation phase after an initial discovery phase. The cohorts investigated reflect a general population but included also women with a high familial risk of breast cancer and BRCA1-mutation carriers (4 benign and 4 malignant samples). The use of pre-treatment breast cancer samples enabled a comparison of miR expression levels without a possible influence of (systemic) therapy.

Limitations of the current study may include the pooled sample-design of the discovery phase. This could have limited the identification of candidate miRs for use in the validation phase. Furthermore, subgroups analyses in the validation cohort were performed with a limited patient number. The relative low expression of *hsa-miR-652-5p* as compared to other miRs (–0.25) in the validation cohort limits the use of the miR as a potential biomarker due to an increased risk of measurements errors influencing the diagnostic accuracy. Given that only *hsa-miR-652-5p* showed an differential expression in both cohorts the diagnostic accuracy for this single miR was not evaluated.

Unexpectedly, when evaluating the results of the unsupervised hierarchical clustering in the discovery phase, benign samples of BRCA1-mutation carriers clustered with the malignant samples. A differential expression for two other miRs (*hsa-miR-185-5p* and *hsa-miR-675-5p*) was additionally found when comparing BRCA1 mutant versus wildtype cases. These results and the differential expression as found by Erturk and colleagues [32, 33] emphasize the need for validation of miR biomarkers as a screening tool in well-balanced cohorts including BRCA1-mutation carriers, especially since in the general population individuals may be unaware of their BRCA-mutation status. This underlines the need for well-balanced cohorts. In this field of research it is of utmost importance that expression data is confirmed in other cohorts before these miRs are used to guide screening and treatment. Furthermore, differences in measurement techniques and normalization methodologies should be minimized enabling an adequate comparison and thus meaningful validation.

## METHODS

### Patients

After informed consent, pre-treatment blood samples were obtained of patients with benign breast disease and those with breast cancer at Erasmus MC, the university hospital in Rotterdam, the Netherlands. This study was approved by the institutional review board (Medical Ethical committee number; MEC-2005-002). Following inclusion 114 samples were separated over the discovery cohort (*n* = 30) and the validation cohort (*n* = 84) dividing the benign and malignant cases. At time of blood sampling patients were allocated to the benign or malignant group. Patients were included only if the diagnosis was histologically proven (if applicable) and long-term follow-up was available. Sufficient plasma (more than 500 μL) had to be available. Patients with a previous history of invasive carcinoma, patients for whom less plasma was available and breast cancer patients with metastasis at time of diagnoses were excluded. Male patients were excluded. Patients who were allocated to the benign group but turned out to have malignant disease during follow-up were additionally excluded in this study. Survival status and recurrence status was updated regularly using patients’ files. Last follow-up date was June 13th 2017.

### Samples

Blood was obtained at the outpatient laboratory of the Erasmus MC. Following venous blood withdrawal (4 tubes per patients), samples were directly divided over micronics tubes (1 mL) separating whole blood, serum and plasma and stored in the freezer (–80°C). Prior to defrosting, dithiothreitol (DTT 5 mM final) was added to the plasma to prevent degradation of nucleic acids during defrosting and handling. Total RNA was isolated from 200 uL plasma with the Norgen Total RNA Purification Kit as recommended by the manufacturer (Norgen Biotek Corp., Canada), adjusted to 43% ethanol during isolation to optimize the miRNA recovery yield.

For the discovery phase, 12 samples were prepared, each sample containing a pool of 2 to 3 plasma RNA aliquots of patients with similar clinical characteristics (Supplementary Table 3). For the validation phase, only individual samples were used.

To ensure that good quality RNA was used only, all RNA samples were checked according to two sample quality control steps. First, 3 uL (5%) of the isolated total RNA was used in a multiplex RT-qPCR pre-screen with 8 validated Taqman MicroRNA Assays (ThermoScientific, The Netherlands). The median value of 5 stable-expressed reference miRs (Figure 2 and Supplementary Table 4) included in this 8-miR pre-screen panel served as a reference value by applying a cut-off value of 20 qPCR cycles. Second, haemolysis miR (Figure 2) expression was evaluated. The ratio of *miR-451* over *miR-23a* was used to evaluate the possible presence of haemolysis in the samples as described previously [22]. As we had the median of 25 miRs (Figure 2) available quantified by RT-qPCR, we decided to use the more robust 25 miR median normalized *miR-451* levels in the final analysis. Samples were excluded from the final analysis if a median normalized *miR-451* value > 6.0 was measured.

### Discovery phase

#### miR profiling

Pooled discovery samples containing either 400 or 600 μL plasma were shipped to TATAA (TATAA BioCenter, Sweden) for 3D-Gene miRNA profiling (Toray Industries, Japan). The 3D-Gene DNA chips contain 2,000 human miRNAs selected from database mirBase release 19.0. The chips were processed according to the standard protocol of the manufacturer (http://www.3d-gene.com/en/).

### Data processing

Analysing the microarray data, the presence or absence of a signal was calculated as follows: spots with intensities higher than the background average + 2-standard deviations were considered “present”. The background average was subtracted from the signal intensities to give the background subtracted values, after which the median signal intensity was calculated. Finally, a normalisation factor was calculated as: 25 divided by the median signal intensity of all background-subtracted data, after which the background subtracted data were multiplied by this normalisation factor. Next, only miRs that were detectable in at least 20% of the groups (Supplementary Table 3) and for which validated Taqman assays were expected to be available [*miR-1* to *miR-700*] were selected for further analysis in the unsupervised analysis (Supplementary Table 4). GeNorm and NormFinder, both present in the software package of GenEx v.6.1 from MultiD, were used to identify the most stable expressed miRs to uniformly normalise the microarray and Taqman reverse transcriptase polymerase chain reaction (RT-qPCR) data. For the microarray data, after background subtraction, thus identified 5 reference miRs (Figure 2) were used for normalizing the RT-qPCR data before identifying the differentially expressed miRs.

Apart from the miR expression present in the unsupervised analysis, 11 additional literature-based miRs (Figure 2 and Supplementary Table 3) previously reported to differentiate benign from malignant breast disease were selected [15–20].

### Validation phase

#### miR profiling

Twenty-five miRs (20 selected miRs and 5 reference miRs) were quantified in the validation cohort by RT-qPCR using validated Taqman mature MicroRNA Assays (ThermoScientific) (Supplementary Table 4). To enable accurate and reliable quantification of multiple miRs in small RNA samples, an extended multiplex RT reaction (40 cycles) was performed as described by the manufacturer for their custom Taqman Array MicroRNA fluidic cards. This was followed by 15 PCR rounds of pre-amplification (PreAmp mastermix from ThermoScientific) and 40 rounds of PCR for quantification [14]. The performance of the RT-qPCR assays was checked using a serially diluted human breast cancer cell line control sample. The control consisted of total RNA isolated from different breast cancer cell lines to ensure all miRs were present in this control sample. A second check was performed with a cell line control sample containing total RNA from the same cell lines, this time however isolated from FFPE to evaluate the performance of the assays with degraded RNA. Only Taqman miRNA assays that could amplify miR targets homogeneously in a multiplexed reverse transcriptase setting within both serially diluted control samples were used for sample validation. This was defined as a specific, with 90-110% efficiency measured, linearly amplified end product [23].

### Data processing

The median of all 25 miRs selected showed the lowest M-value and smallest inter- and intragroup variation and was therefore used as reference value to normalise RT-qPCR data in the validation phase (Supplementary Table 4).

MiR expression levels were compared in the validation cohort between 1) benign versus malignant samples and 2) non-metastatic versus metastatic breast cancer samples. Secondly the expression of the differentially expressed miRs were evaluated according to patients’ age (≤50 versus >50), familial breast cancer (no versus yes), tumour stage (T1 versus >T1), nodal stage (N0 versus N+), hormone receptor status, Her2Neu receptor status and triple negative status (negative for ER, PR and Her2Neu). Hormonal receptor status was considered positive in case of ≥10% nuclear staining of the oestrogen receptor and/or progesterone receptor. Based on the unsupervised clustering of miRs in the discovery cohort, separate analyses were performed with the *BRCA1*-mutation carrier samples (due to low numbers, combined from both the discovery and validation phase). Benign and malignant samples from *BRCA1* carriers were compared to respectively wildtype benign and malignant samples of the validation cohort.

### Statistics

All data were analysed with SPSS version 21 (IBM). To compare groups with numerical variables, the Mann–Whitney *U* test was used. The Fisher's exact test was used to compare groups in 2×2 contingency tables. For 2×3 and 2×4 tables, the Freeman-Halton extension of the Fisher's exact probability test was used. To correct for multiple comparisons *P*-values were adjusted for type I errors by using the Benjamini-Hochberg false discovery rate (FDR) method at a cut off of 10% [24]. All *P*-values are 2-sided and *P* < 0.05 was considered statistically significant.

## CONCLUSIONS

With *hsa-miR-652-5p* showing promising results as a potential early breast cancer biomarker and multiple studies showing discriminative blood-born miR expression for the detection of breast cancer, future research should focus on validating previously detected miRs and preferably miR-signatures in cohorts representative of the screening population. This could lead to sufficient validation of a miR-signature serving as biomarker and thereby enabling improved early breast cancer as well as early metastasis detection.

## SUPPLEMENTARY MATERIALS FIGURES AND TABLES







## Abbreviations

FDR: False-Discovery Rate
FFPE: Formalin-Fixed Paraffin-Embedded
HER2: Human Epidermal growth factor Receptor 2
IQR: Interquartile Range
MIR: MicroRNA
MRNA: Messenger RNA
PCR: Polymerase Chain Reaction
RT-qPCR: Quantative Reverse Transcription PCR
TNBC: Triple Negative Breast Cancer
