# Correction: MicroRNA expression in pre-treatment plasma of patients with benign breast diseases and breast cancer

**DOI:** 10.18632/oncotarget.25979

**Published:** 2018-08-10

**Authors:** Mirelle Lagendijk, Sepideh Saadatmand, Linetta B. Koppert, Madeleine M.A. Tilanus-Linthorst, Vanja de Weerd, Raquel Ramírez-Moreno, Marcel Smid, Anieta M. Sieuwerts, John W.M. Martens

**Affiliations:** ^1^ Department of Surgical Oncology, Erasmus MC Cancer Institute, EA 3075, Rotterdam, The Netherlands; ^2^ Department of Medical Oncology, Erasmus MC Cancer Institute, EA 3075, Rotterdam, The Netherlands; ^3^ Cancer Genomics Centre Netherlands, Erasmus University MC, CN 3015, Rotterdam, The Netherlands

**This article has been corrected:** The correct author name is given below:

**Sepideh Saadatmand**

Original article: Oncotarget. 2018; 9:24335-24346. https://doi.org/10.18632/oncotarget.25262

