# Supplementary material for: MicroRNA expression in pre-treatment plasma of patients with benign breast diseases and breast cancer
Source: Oncotarget. 2018 May 11;9(36):24335–46. doi: 10.18632/oncotarget.25262 (PMC5966243; doi:10.18632/oncotarget.25262)
Supplement: Supplementary file 1 [file oncotarget-09-24335-s001.pdf]

MicroRNA expression in pre-treatment plasma of patients with benign breast diseases and breast cancer

SUPPLEMENTARY MATERIALS

Supplementary Table 1: Expression levels of 30 miRs in the discovery cohort. See Supplementary_Table 1

Supplementary Table 2: Overview of literature for literature-based miRs. See Supplementary_Table 2

Supplementary Table 3: Subgroups in the discovery cohort based on pooled samples

Pooled samples	Pool		Clinical group
Benign, papiloma	1	A	**A = wildtype BRCA1 benign**<br>**Group A consists of pool 1 + 2 + 3.**
Benign, fibroadenoma (aged ≤50 years)	2	A	
Benign (aged > 50 years)	3	A	
Benign samples of BRCA1 mutation carriers	4		**B = mutant BRCA1 benign (pool 4)**
Malignant, N0, HR positive, Her2Neu negative, and chemotherapy naïve	5		**tC = malignant**
Malignant, N0/N1, HR negative and Her2Neu negative	6		
Malignant, T2-T4, HR positive, Her2Neu negative with chemotherapy (aged ≤50 years)	7		
Malignant, T2-T4, HR positive. Her2Neu negative and chemotherapy naïve (aged >50years)	8		
Malignant, HR positive and Her2Neu positive	9		
Metastatic breast cancer, T1a-T1b, N0, HR positive and Her2Neu negative (aged ≤50 years)	10		
Metastatic breast cancer, N+, HR positive and Her2Neu negative (aged > 50 years)	11		
Metastatic breast cancer, HR negative and Her2Neu negative	12		

'N' = nodal stage. 'HR' = hormonal receptor status. 'Her2Neu' = Her2Neu receptor status.

Supplementary Table 4: GeNorm and NormFinder results for the discovery (*n* = 29) and validation (*n* = 79) samples

GeNorm		NormFinder	intergroup variation		intragroup variation	
			benign	malignant+ metastatic	benign	malignant+ metastatic
**Gene name**	***M*-Value**	**Gene name**	**SD**	**SD**	**SD**	**SD**
**Discovery Phase micro-array [*n* = 29]**						
hsa-miR-125a-5p	0.51	*hsa-miR-125a-5p*	–0.18	0.18	0.26	0.07
hsa-miR-197-3p	0.34	*hsa-miR-197-3p*	–0.16	0.16	0.10	0.02
hsa-miR-326	0.43	*hsa-miR-326*	–0.26	0.26	0.06	0.15
hsa-miR-423-5p	0.42	*hsa-miR-423-5p*	0.08	–0.08	0.24	0.18
hsa-miR-532-3p	0.34	*hsa-miR-532-3p*	–0.19	0.19	0.18	0.07
hsa-miR-188-5p	0.40	*hsa-miR-188-5p*	–0.01	0.01	0.04	0.01
**Validation phase RT-qPCR [*n* = 79]**						
median_25miR_panel	0.50	*median_25miR_panel*	0.02	–0.02	0.08	0.05
median_5miR_panel	0.60	*median_5miR_panel*	–0.06	0.06	0.30	0.25
Cq_miR_103	0.47	*Cq_miR_103*	0.14	–0.14	0.25	0.39
Cq_miR_107	0.47	*Cq_miR_107*	0.10	–0.10	0.18	0.29
Cq_miR_423_5p	0.51	*Cq_miR_423_5p*	0.14	–0.14	0.30	0.24
Cq_miR_142_3p_24	0.52	*Cq_miR_142_3p_24*	0.04	–0.04	0.19	0.21
Cq_miR_185	0.54	*Cq_miR_185*	0.13	–0.13	0.06	0.36
Cq_miR_21	0.56	*Cq_miR_21*	0.02	–0.02	0.14	0.09
Cq_miR_191	0.58	*Cq_miR_191*	0.10	–0.10	0.44	0.30
Cq_miR_532_3p	0.59	*Cq_miR_532_3p*	–0.03	0.03	0.23	0.11
Cq_miR_197	0.62	*Cq_miR_197*	–0.13	0.13	0.30	0.38
Cq_miR_195	0.63	*Cq_miR_195*	0.07	–0.07	0.27	0.11
Cq_miR_125a_5p	0.66	*Cq_miR_125a_5p*	0.04	–0.04	0.52	0.58
Cq_miR_20b	0.69	*Cq_miR_20b*	0.07	–0.07	0.24	0.63
Cq_miR_let7b	0.73	*Cq_miR_let7b*	–0.25	0.25	0.74	0.48
Cq_miR_145	0.77	*Cq_miR_145*	0.00	0.00	0.69	1.14
Cq_miR_652_5p	0.80	*Cq_miR_652_5p*	0.32	–0.32	0.71	1.44
Cq_miR_29b	0.84	*Cq_miR_29b*	0.18	–0.18	0.62	1.44
Cq_miR_18b	0.88	*Cq_miR_18b*	–0.09	0.09	1.55	0.69
Cq_miR_375	0.93	*Cq_miR_375*	–0.10	0.10	1.34	1.37
Cq_miR_451	0.99	*Cq_miR_451*	0.11	–0.11	1.63	2.27
Cq_miR_675	1.06	*Cq_miR_675*	–0.10	0.10	1.76	3.33
Cq_miR_135a[*]	1.14	*Cq_miR_135a*[*]	–0.30	0.30	1.87	4.70
Cqa_miR_23a[*]	1.24	*Cqa_miR_23a*[*]	–0.37	0.37	2.60	7.44
Cq_miR_202	1.34	*Cq_miR_202*	–0.05	0.05	1.29	9.10
Cq_miR_326	1.44	*Cq_miR_326*	0.69	–0.69	4.26	8.51
Cq_miR_382star	1.56	*Cq_miR_382star*	–0.70	0.70	5.14	12.95

Of the 6 stable expressed miRs identified in the micro-array data all except *hsa-miR-188-5p* could be measured reliably by RT-qPCR in the validation set. As the median value of the 25 miR panel with an *M*-value of 0.50 versus 0.60 for the remaining 5-miR reference miR panel was identified as the most stable marker for our analysis, we normalized our data on the median level of all 25 miRs that could be quantified reliably.

Also when analyzed separately for benign and malignant/metastatic with NormFinder, the median of the 25 miR panel had the lowest variability.
