# Supplementary material for: MicroRNA expression in pre-treatment plasma of patients with benign breast diseases and breast cancer
Source: Oncotarget. 2018 May 11;9(36):24335–46. doi: 10.18632/oncotarget.25262 (PMC5966243; doi:10.18632/oncotarget.25262)
Supplement: Supplementary file 2 [file oncotarget-09-24335-s002.docx]

**Supplementary files**

**Supplementary Table S1. Expression levels of 30 miRs in the discovery cohort.**

|  | *miR-name** | Assay ID ThermoFisher | median 6refmiRnorm  1,2,3^§^ [A] | median 6refmiRnorm  1,2,3,4^§^ [B] | median 6refmiRnorm 5-12^§^ [C] | B/A | Fold Change group A | Fold Change group B | group A vs C  P-value# | group B vs C  P-value# |
| --- | --- | --- | --- | --- | --- | --- | --- | --- | --- | --- |
| Reference miRs | *hsa-miR-125a-5p* | 2198 | 0.6 | 0.6 | 0.7 | 1.1 | 0.88 | 0.94 | 1.0E+00 | 1.0E+00 |
|  | *hsa-miR-197-3p* | 497 | 1.1 | 1.1 | 1.2 | 1.0 | 0.97 | 0.97 | 6.8E-01 | 5.0E-01 |
|  | *hsa-miR-423-5p* | 2340 | 0.7 | 0.7 | 0.5 | 1.0 | 1.43 | 1.36 | 5.4E-01 | 5.0E-01 |
|  | *hsa-miR-326* | 542 | 2.5 | 2.9 | 3.2 | 1.1 | 0.79 | 0.89 | 1.5E-01 | 3.1E-01 |
|  | *hsa-miR-532-3p* | 2355 | 0.9 | 0.9 | 0.8 | 1.0 | 1.14 | 1.15 | 3.1E-01 | 2.3E-01 |
|  | *hsa-miR-188-5p* | 2320 | 1.7 | 1.9 | 2.3 | 1.2 | 0.73 | 0.84 | 6.6E-02 | 4.0E-01 |
|  |  |  |  |  |  |  |  |  |  |  |
| Haemolyse marker | *hsa-miR-451a* | 1141 | 3.3 | 4.0 | 2.9 | 1.2 | 1.14 | 1.38 | 1.0E+00 | 8.7E-01 |
|  | *hsa-miR-23a-5p* | 2439 | 1.6 | 1.8 | 1.7 | 1.1 | 0.94 | 1.07 | 1.0E+00 | 8.7E-01 |
|  |  |  |  |  |  |  |  |  |  |  |
| Candidate miRs* | *hsa-miR-135a-3p* | 2232 | 7.6 | 7.6 | 17.9 | 1.0 | 0.42 | 0.43 | 1.4E-02 | 2.7E-02 |
|  | *hsa-miR-185-5p* | 2271 | 0.8 | 1.3 | 3.0 | 1.5 | 0.28 | 0.43 | 2.5E-02 | 1.7E-02 |
|  | *hsa-miR-652-5p* | 472084_mat | 15.4 | 15.3 | 26.4 | 1.0 | 0.58 | 0.58 | 6.6E-02 | 2.7E-02 |
|  | *hsa-miR-675-5p* | 2005 | 8.8 | 9.1 | 18.8 | 1.0 | 0.47 | 0.48 | 1.4E-02 | 2.7E-02 |
|  | *hsa-miR-345-3p* | 474987_mat | 1.7 | 1.8 | 4.0 | 1.1 | 0.43 | 0.46 | 1.4E-02 | 1.1E-02 |
|  | *hsa-miR-513b* | 2757 | 0.7 | 0.8 | 1.6 | 1.1 | 0.42 | 0.48 | 1.4E-02 | 1.7E-02 |
|  | *hsa-miR-642a-3p* | 474715_mat | 33.3 | 35.4 | 58.6 | 1.1 | 0.57 | 0.60 | 2.5E-02 | 1.7E-02 |
|  |  |  |  |  |  |  |  |  |  |  |
| BRCA1 specific** | *hsa-miR-20b-5p* | 1014 | 0.3 | 1.0 | 2.0 | 2.9 | 0.17 | 0.51 | 3.6E-01 | 4.4E-01 |
|  | *hsa-miR-21-5p* | 397 | 2.1 | 4.4 | 4.8 | 2.1 | 0.43 | 0.90 | 1.0E+00 | 1.0E+00 |
|  | *hsa-miR-103a-3p* | 439 | 0.4 | 1.1 | 2.1 | 2.6 | 0.20 | 0.54 | 2.2E-01 | 3.1E-01 |
|  | *hsa-miR-142-3p* | 464 | 0.8 | 2.0 | 3.1 | 2.6 | 0.25 | 0.65 | 1.0E+00 | 7.3E-01 |
|  |  |  |  |  |  |  |  |  |  |  |
| Literature-based miRs | *hsa-miR-107* | 443 | 0.9 | 1.3 | 1.7 | 1.4 | 0.51 | 0.74 | 1.0E+00 | 1.0E+00 |
|  | *hsa-miR-145-5p* | 2278 | 0.0 | 0.3 | 0.6 | 32.6 | 0.02 | 0.57 | 1.0E-01 | 1.7E-01 |
|  | *hsa-miR-18b-5p* | 2217 | 0.6 | 0.6 | 0.7 | 1.0 | 0.81 | 0.84 | 5.4E-01 | 6.1E-01 |
|  | *hsa-miR-191-5p* | 2299 | 0.8 | 1.0 | 1.3 | 1.3 | 0.60 | 0.77 | 5.4E-01 | 6.1E-01 |
|  | *hsa-miR-195-5p* | 494 | 0.4 | 0.5 | 0.6 | 1.3 | 0.62 | 0.82 | 3.1E-01 | 4.0E-01 |
|  | *hsa-miR-202-3p* | 2363 | 0.7 | 1.0 | 0.5 | 1.3 | 1.40 | 1.85 | 1.5E-01 | 6.2E-02 |
|  | *hsa-miR-375* | 564 | 18.4 | 17.4 | 12.4 | 0.9 | 1.48 | 1.40 | 3.1E-01 | 3.1E-01 |
|  | *hsa-miR-382-3p* | 1354 | 0.0 | 0.0 | 0.4 | 1.0 | 0.02 | 0.02 | 1.3E-02 | 4.0E-02 |
|  | *hsa_let-7b* | 378 | 1.7 | 1.7 | 2.2 | 1.0 | 0.78 | 0.80 | 4.1E-01 | 6.1E-01 |
|  | *hsa_miR-29b* | 413 | 0.0 | 0.0 | 0.6 | 1.0 | 0.02 | 0.02 | 2.8E-02 | 6.9E-02 |
|  | *hsa-miR-92a-2-5p* | 2138 | 28.1 | 28.3 | 37.2 | 1.0 | 0.76 | 0.76 | 4.1E-02 | 6.2E-02 |

^§^ Pooled samples analysis, groups are specified in Supplementary Table S1.

^#^ Mann-Whitney U-test. *Only miRs named <700 were included to ensure available Taqman assays.

.* We selected 7 candidate miRs with a reference normalised value >1 in group C to ensure measurable levels, a P-value<0.05 and a fold-change for group B / group C< 0.06. 4 out of the 7 selected miRs passed our QC.

** Selected based on at least 2-fold higher in the benign BRCA1 group.
