# Supplementary material for: MicroRNA expression in pre-treatment plasma of patients with benign breast diseases and breast cancer
Source: Oncotarget. 2018 May 11;9(36):24335–46. doi: 10.18632/oncotarget.25262 (PMC5966243; doi:10.18632/oncotarget.25262)
Supplement: Supplementary file 3 [file oncotarget-09-24335-s003.docx]

**Supplementary Table S2. Overview of literature for literature-based miRs.**

| **Literature-miR** | **Study by author** | **Comparisons (number of samples)** | **Regulation (Fold change if available)** | **Reference miR(s)** |
| --- | --- | --- | --- | --- |
| *Hsa-miR-18b* | Kleivi Sahlberg, Bottai [1] | **Blood samples:** -Non-relapsing (20) and relapsing TNBC (20).  -Healthy control (30) versus TNBC patients (40).  -ER-positive breast cancer patients (33) versus TNBC patients (40).  -Healthy controls versus ER-positive breast cancer patients | -Upregulation in the relapsing samples.  -Upregulation in the TNBC samples  -Upregulation in the TNBC samples.  -No significant differences, | *Hsa-miR-32 and hsa-miR-101* |
| *Has-miR-20* | Si, Sun [2] | **Blood samples:** Healthy controls (20) versus breast cancer patients (100). | Downregulation in breast cancer samples. | *Hsa-miR-16* |
|  | Wang and Zhang [3] | **Blood samples:** Healthy controls (39) versus Breast cancer (50) + Gastric cancer (30) + Lung cancer (31) + Oesophageal cancer (31) and Colorectal cancer (32) | Downregulation in all cancer samples. | *Hsa-miR-16* |
| *Hsa-miR-29b* | Shaker, Maher [4] | **Blood samples:** Healthy controls (30) non-metastatic breast cancer (80) and metastatic breast cancer patients (20) | Upregulation in breast cancer patients |  |
|  | Li, Guo [5] | **Colorectal tissue:** age and gender matched control samples (400) versus 200 colorectal tissues of colorectal carcinomas | Downregulation in colorectal tissues | Spike-U16 |
| *Hsa-miR-92a-2-5p* | Si, Sun [2] | **Blood samples:** Healthy controls (20) versus breast cancer patients (100). | Downregulation in breast cancer samples. | *Hsa-miR-16* |
|  | Chan, Liaw [6] | **Blood samples:** Healthy controls (101) versus breast cancer patients (132). | Upregulation in breast cancer patients (1.34) | *Hsa-miR-103 and hsa-miR-191* |
| *Hsa-miR-107* | Kleivi Sahlberg, Bottai [1] | **Blood samples:** Non-relapsing (20) and relapsing TNBC patients (20) | Upregulation in the relapsing samples. | *Hsa-miR-32 and hsa-miR-101* |
| *Hsa-miR-145-5p* | Chan, Liaw [6] | **Tissue samples:** Adjacent normal tissue (23) versus breast cancer tissue (31). | Downregulation in breast cancer tissue (2.48). | *Hsa-miR-103 and hsa-miR-191* |
|  | Mar-Aguilar, Mendoza-Ramirez [7] | **Blood samples:** Health controls (10) versus breast cancer patients (61). | Upregulation in breast cancer patients. | 18SRNA |
| *Hsa-miR-191* | Mar-Aguilar, Mendoza-Ramirez [7] | **Blood samples:** Health controls (10) versus breast cancer patients (61). | Upregulation in breast cancer patients. | 18SRNA |
| *Hsa-miR-195* | Heneghan, Miller [8] | **Blood samples: -**63 healthy age-matched control patients versus 163 cancer patients*  -63 healthy age-matched controls versus 83 cancer patients. | -Upregulation in cancer patients.  -Upregulation in breast cancer patients (25). | *Hsa-miR-16* |
| *Hsa-miR-202* | Schrauder, Strick [9] | **Blood samples:** 24 age-matched healthy controls versus 24 breast cancer patients. | Downregulation in breast cancer patients (2.08). | *Hsa-miR-16* |
| *Hsa-miR-375* | Madhavan, Zucknick [10] | **Blood samples:** 76 healthy controls versus 133 metastatic breast cancer patients. | Upregulation in CTC-positive metastatic breast cancer patients. | Cell *miR-39* |
| *Hsa-miR-382* | Mar-Aguilar, Mendoza-Ramirez [7] | **Blood samples:** Health controls (10) versus breast cancer patients (61). | Upregulation in breast cancer patients. | 18SRNA |
| *Let-7b* | Chan, Liaw [6] | **Blood samples:** Healthy controls (101) versus breast cancer patients (132). | Upregulation in breast cancer patients (0.85). | *hsa-miR-103* and *hsa-miR-191* |
|  | Freres, Wenric [11] | **Blood samples:** Controls (133), primary breast cancer patients (149), metastatic breast cancer (31), breast cancer in remission (35) and gynaecologic cancer (30) | Upregulation in primary breast cancer and metastatic breast cancers (0.7 and 0.8 respectively) | Normalisation on 50miRs most regularly expressed |
|  | Joosse, Muller [12] | **Blood samples:** Healthy woman (37), benign breast disease (26) and breast cancer patients (102) | Upregulation in benign breast disease and further downregulation in breast cancer patients |  |

TNBC = triple negative breast cancer, ER = Oestrogen receptor

*83 breast cancer, 30 colon cancer, 20 prostate cancer, 20 renal cell carcinoma and 10 malignant melanoma patients.

**Reference list Supplementary Table S2.**

1. Kleivi Sahlberg K, Bottai G, Naume B, Burwinkel B, Calin GA, Borresen-Dale AL and Santarpia L. A serum microRNA signature predicts tumor relapse and survival in triple-negative breast cancer patients. Clin Cancer Res. 2015; 21(5):1207-1214.

2. Si H, Sun X, Chen Y, Cao Y, Chen S, Wang H and Hu C. Circulating microRNA-92a and microRNA-21 as novel minimally invasive biomarkers for primary breast cancer. J Cancer Res Clin Oncol. 2013; 139(2):223-229.

3. Wang B and Zhang Q. The expression and clinical significance of circulating microRNA-21 in serum of five solid tumors. J Cancer Res Clin Oncol. 2012; 138(10):1659-1666.

4. Shaker O, Maher M, Nassar Y, Morcos G and Gad Z. Role of microRNAs -29b-2, -155, -197 and -205 as diagnostic biomarkers in serum of breast cancer females. Gene. 2015; 560(1):77-82.

5. Li L, Guo Y, Chen Y, Wang J, Zhen L, Guo X, Liu J and Jing C. The Diagnostic Efficacy and Biological Effects of microRNA-29b for Colon Cancer. Technol Cancer Res Treat. 2016; 15(6):772-779.

6. Chan M, Liaw CS, Ji SM, Tan HH, Wong CY, Thike AA, Tan PH, Ho GH and Lee AS. Identification of circulating microRNA signatures for breast cancer detection. Clin Cancer Res. 2013; 19(16):4477-4487.

7. Mar-Aguilar F, Mendoza-Ramirez JA, Malagon-Santiago I, Espino-Silva PK, Santuario-Facio SK, Ruiz-Flores P, Rodriguez-Padilla C and Resendez-Perez D. Serum circulating microRNA profiling for identification of potential breast cancer biomarkers. Dis Markers. 2013; 34(3):163-169.

8. Heneghan HM, Miller N, Lowery AJ, Sweeney KJ, Newell J and Kerin MJ. Circulating microRNAs as novel minimally invasive biomarkers for breast cancer. Ann Surg. 2010; 251(3):499-505.

9. Schrauder MG, Strick R, Schulz-Wendtland R, Strissel PL, Kahmann L, Loehberg CR, Lux MP, Jud SM, Hartmann A, Hein A, Bayer CM, Bani MR, Richter S, Adamietz BR, Wenkel E, Rauh C, et al. Circulating micro-RNAs as potential blood-based markers for early stage breast cancer detection. PLoS One. 2012; 7(1):e29770.

10. Madhavan D, Zucknick M, Wallwiener M, Cuk K, Modugno C, Scharpff M, Schott S, Heil J, Turchinovich A, Yang R, Benner A, Riethdorf S, Trumpp A, Sohn C, Pantel K, Schneeweiss A, et al. Circulating miRNAs as surrogate markers for circulating tumor cells and prognostic markers in metastatic breast cancer. Clin Cancer Res. 2012; 18(21):5972-5982.

11. Freres P, Wenric S, Boukerroucha M, Fasquelle C, Thiry J, Bovy N, Struman I, Geurts P, Collignon J, Schroeder H, Kridelka F, Lifrange E, Jossa V, Bours V, Josse C and Jerusalem G. Circulating microRNA-based screening tool for breast cancer. Oncotarget. 2016; 7(5):5416-5428.

12. Joosse SA, Muller V, Steinbach B, Pantel K and Schwarzenbach H. Circulating cell-free cancer-testis MAGE-A RNA, BORIS RNA, let-7b and miR-202 in the blood of patients with breast cancer and benign breast diseases. Br J Cancer. 2014; 111(5):909-917.
